# The relationship between fall incidents and place of birth in residential aged care facilities: a retrospective longitudinal cohort study

**DOI:** 10.1186/s12877-023-03954-7

**Published:** 2023-04-28

**Authors:** Guogui Huang, Nasir Wabe, Magdalena Z. Raban, Karla L. Seaman, Sandun Malpriya Silva, Johanna I. Westbrook

**Affiliations:** Centre for Health Systems and Safety Research, Macquarie University, North Ryde, NSW 2109 Macquarie, Australia

**Keywords:** Falls incidence, Fall risk, Place of birth, Cultural and linguistical diversity, Aged care

## Abstract

**Background:**

Older populations in residential aged care facilities (RACFs) in many immigrant-receiving countries are now being increasingly culturally and linguistically diverse (CALD). CALD populations require tailored social and health services to support their needs and improve health outcomes. Falls among the elderly are common and can have significant health and psychosocial consequences. There is some evidence to suggest that country of birth may influence risk of falls among older people, but such evidence has been scarce. This study aimed to determine the association between place of birth and the incidence of falls in RACFs.

**Methods:**

Routinely collected incident data relating to 5,628 residents aged ≥ 65 years in 25 RACFs in Sydney, New South Wales, Australia were used. RACF residents were classified into two groups, Australia-born (N = 4,086) and overseas-born (N = 1,542). Overseas-born RACF residents were further categorised into two subgroups: overseas-English-speaking-country (N = 743) and overseas-non-English-speaking-country (N = 799). Outcomes measures were rate of all falls, injurious falls and falls requiring hospitalisation. Multilevel binary negative regression was used to examine the relationship between fall risk and place of birth.

**Results:**

Incidence rates of all falls, injurious falls and falls requiring hospitalisation were 8.62, 3.72 and 1.07 incidents per 1,000 resident days, respectively, among the Australia-born RACF residents, but were higher at 11.02, 4.13 and 1.65, respectively, among the overseas-born RACF residents. Within those born overseas, fall rates were higher among the overseas-non-English-speaking-country-born residents (11.32, 4.29 and 2.22, respectively) than those overseas-English-speaking-country-born (10.70, 3.96 and 1.05, respectively). After controlling for confounders, the overseas-born RACF residents overall experienced a higher risk of all three types of falls (incidence rate ratios: [IRR] = 1.278, 95% confidence interval [CI] = 1.131, 1.443; injurious falls: IRR = 1.164 [95% CI = 1.013, 1.338]; falls requiring hospitalisation: IRR = 1.460 [95% CI = 1.199, 1.777]) than the Australia-born RACF residents. Among the overseas-born RACF residents, males, respite residents and those overseas-non-English-speaking-country-born experienced higher rates of falls.

**Conclusions:**

Fall incidence in RACFs varies significantly by place of birth. With increasingly diverse RACF populations, fall intervention and prevention programs should consider cultural and linguistical backgrounds of RACF residents. Greater attention to understand the mechanisms for the differences by place of birth in risk profiles is warranted.

**Supplementary Information:**

The online version contains supplementary material available at 10.1186/s12877-023-03954-7.

## Introduction

Falls and fall-related injuries among older populations are a prominent concern globally. Approximately 28–35% of people aged 65 and older in the community [1, 2] and more than 50% of those living in residential aged care facilities (RACFs) (i.e., nursing homes and long-term care facilities) [3] fall annually. Devastating clinical consequences of falls include various injuries, multisite chronic pain, immobilisation, disability and even death [4–7]. Falls might lead to fear of falling that may prevent older adults from engaging in social activities and adversely affect older people’s quality of life [8, 9]. Falls also cause high financial burdens, with fall-related costs accounting for 1.3–2.3% of national health care expenditure in the United States, 1.4% in the United Kingdom and 1.1% in Australia [10].

Previous studies have identified a range of risk factors of falls, including older age, female gender, declined cognitive and physical functionality, use of psychotropic medication and fall history [1, 2, 5, 6, 11]. Recent studies have shown that place of birth may also affect the risk of falls in later life, but with mixed findings reported. One research in Australia demonstrated that the fall incidence of Italy-born community-dwelling men aged 70 years and older was 43% lower than that of similarly-aged Australia-born men living in the community (incidence rate ratios [IRR] = 0.57, 95% confidence interval [CI] = 0.39–0.85) over a four-month study period [12]. Similarly, evidence from a report of the Australian Institute of Health and Welfare revealed that the rate of fall-related hospitalisation among older adults (≥ 65) during 2001–2003 for migrants from New Zealand, the United Kingdom, Ireland, the United States, Canada and South Africa and migrants from other countries, numbered at 2,070.3 and 1,788.6 per 100,000 population, respectively, both significantly lower than that of the Australia-born population (i.e., 2,498.1 per 100,000 population) [13]. Similar findings were documented in Sweden, showing a relatively low fall rate in Swedish counties with a high proportion of overseas-born older adults [14]. In contrast, a population-based study conducted in the communities of South Australia reported no difference in fall risk between Australia-born older adults (≥ 65) and older immigrants from overseas English-speaking countries (odd ratio [OR] = 0.95, 95% CI = 0.76–1.18), though immigrants from non-English-speaking countries had a lower fall risk than the Australia-born older adults (OR = 0.53 [95% CI = 0.38–0.74) [15]. Likewise, a cross-national cohort study indicated that a sample of Australian Chinese aged 70 and older experienced a higher age- and sex-standardised annual fall rate (mean + standard deviation: 0.36 ± 0.80) than the Chinese populations in Hong Kong (0.21 ± 0.57) and Taiwan (0.26 ± 0.47), suggesting an adverse effect of immigration experience and post-immigration environment on the susceptibility to falls [16].

Previous research has demonstrated that the prevalence and consequences of age-related conditions might prevail and pattern differently by country of birth and that such differences may persist despite moving to and residing in another country for many years [17, 18]. Falls among the elderly might also characterise differently by place of birth given the distinguishing life experience of immigrants and varying sociocultural backgrounds by nativity [12, 19]. For example, some Asian immigrants tend to have a lower fall risk, which might be because of their some cultural practices, such as Taichi that is expected to reduce fall risk [20]. Likewise, Caucasians are more likely to fall possibly because of their more frequent participation in outdoor activities that might be linked to a greater likelihood of fall risk [19]. There are also studies demonstrating differences in fall mechanics by race, showing white women tend to fall laterally and sustain a fracture, while black women tend to fall forward and are less likely to sustain a fracture [21]. However, although previous studies have provided important information about the association between falls and place of birth, such evidence has been scarce. Additionally, to the best of our knowledge, there have been no studies on falls and place of birth focused on the RACF setting, where older adults are more likely to fall than those in the community [22]. Our aim was to investigate the relationship between falls and place of birth in Australian RACFs where the population is increasingly culturally and linguistically diverse (CALD) [23]. The new knowledge produced will advance our understanding of the relationship between falls and place of birth among older adults and applied to inform the design of fall prevention programs in RACFs to better support subpopulations at high risk of falls and fall-related consequences.

## Methodology

### Study design

We conducted a retrospective longitudinal cohort study using routinely collected aged care data sourced from 25 RACFs of a large not-for-profit aged care provider in New South Wales, Australia. Routinely-collected data refer to information collected systematically and electronically by aged care providers for clinical and administration purposes on a day-to-day basis. [24] In this study, the data covered a period from 1st July 2014 to 31st December 2019, during which participants could enter, leave and re-enter the facilities at any time. Therefore, one RACF resident might have more than one record of admission during this period.

### Sample

**Fig. 1:**
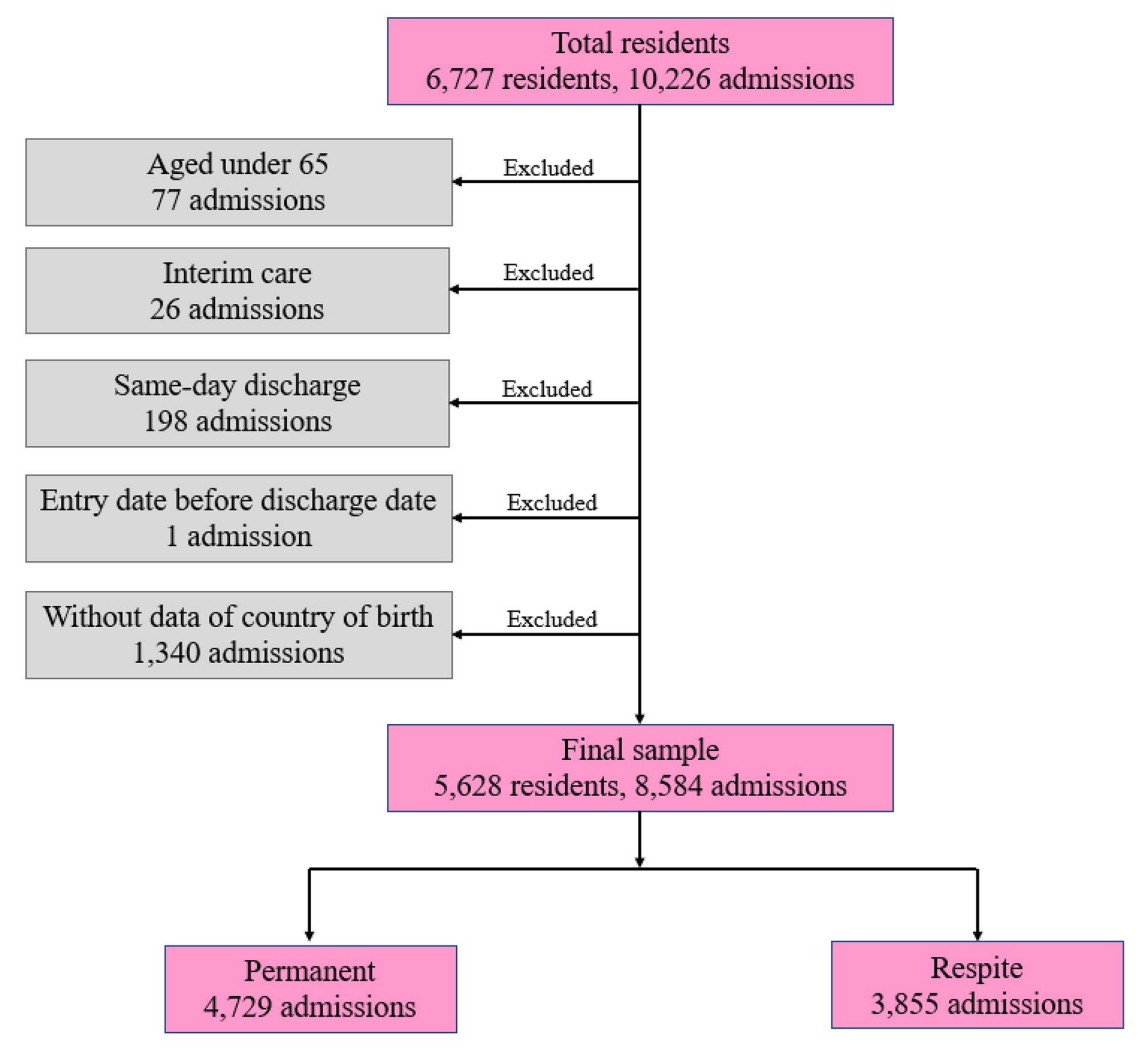
Flow Chart of Sample Selection

Our data contained information of 10,226 admissions from 6,727 residents. We excluded data of admissions in which age of participants was under 65 (N = 77), only interim care was used (N = 26), participants were discharged on the same date as admission (N = 198), participants’ date of entry was erroneously before the departure date (N = 1), or participations’ information of country of birth was missing (N = 1,340). This yielded a final sample of 5,628 residents with 8,584 admissions, of which 4,729 were permanent admissions and 3,855 were respite admissions. The sample selection process is presented in Fig. 1.

### Data sources

We used resident profile data and incident reports. The profile dataset contained information of residents’ demographic characteristics (e.g., year of birth, gender and country of birth), health conditions (e.g., whether having common cormobidities, such as diabetes and hypertension) and admission-related information (e.g., patient ID, facility ID and date of entry/departure). The incident data included all fall events occurring in facilities, including incident date, location (e.g., dining room, resident’s room and shared indoor area) and consequences (e.g., whether results in injuries, location of injuries and if hospitalisation wasd required).

### Outcome measures

We considered three fall outcome groups: all falls, injurious falls and falls requiring hospitalisation. All falls were defined as any fall in the incident dataset regardless of the fall consequences (i.e., causing injuries or requiring hospitalisation); injurious falls were falls that caused any type of injury (e.g., head injury, hip injury or deep issue injury); while falls requiring hospitalisation were those falls resulting in sequalae requiring hospital admission for further assessment and management.

### Measures of place of birth

Two variables of the resident profile dataset, ‘whether Australia-born’ and ‘country of birth if born overseas’, were used to categorise participants as Australia-born or overseas-born. The overseas-born group was further divided into: residents born in an English- or non-English-speaking country based on whether English is listed as an official language in their country of birth [25]. This is because proficiency of the main language used in host society could importantly affect overseas-born individuals’ access to health care services, employability and social capital [26, 27], which are crucial predicators of wellbeing and health outcomes. In addition, English proficiency might also affect overseas-born RACF residents’ daily communication with staff and their involvement in fall prevention and intervention programs, which could affect their fall outcomes.

### Statistical analysis

We calculated fall incidence rates (i.e., per 1,000 resident days). Fall incidence rate for each RACF resident was based on their number of falls occurring during their length of stay. For RACF residents with more than one admission, falls occurring in all the admissions and the lengths of each stay were added up separately. We did not use two commonly used indicators of fall risk, whether an individual suffered a fall or not and the absolute number of falls that the individual experienced [12, 16, 19, 28], given that these two indicators generally require the same length of observation period for each participant for comparison purpose. Therefore, these two indicators were inappropriate in this study due to the varying lengths of stay of different participants.

We first described and compared the average incidence rate of the three types of falls for the Australia-born and the overseas-born RACF residents, and also for the two subgroups of the overseas-born RACF residents. We then conducted multilevel negative binomial regression to examine the association between fall and place of birth, controlling for age, gender, fall history and comorbidity status at time of admission (i.e., dementia, depression, cognitive impairment, anxiety, cerebrovascular accident, diabetes mellitus, visual impairment, delirium and Parkinson’s disease), which have been previously been demonstrated as fall risk factors [5, 6, 11, 29–31]. The multilevel binary negative regression is appropriate for this study given that the dependent variable in our analysis (i.e., fall incidence rate) is an over-dispersed count variable and that participants were organised in two levels (i.e., individual level and facility level). The two-level structure of data means that fall incidents might suffer clustering effect, which refers to the potential correlation between incidents occurring at the same facility. To compare differences in fall incidence rate between two specific groups, we used IRR, calculated as the ratio of the fall incidence rates of the two groups, with the corresponding standard deviation calculated as the square root of the sum of the reciprocals of fall numbers of the two groups. All tests were two-tailed and significance was set as p-value < 0.05. All analyses were conducted using Stata version 17.0 (StataCorp LP, College Station, Texas 77,845 USA).

## Results

### Participant characteristics

As shown in Table 1, 1,896 (33.7%) of participants were men and 1,542 (27.4%) were overseas-born, of which 743 were born in overseas English-speaking countries and 799 were born in overseas non-English-speaking countries. The median age of the whole sample was 86 (interquartile range [IQR] 81–90) years, while the median length of stay was 542 (IQR 69 − 1,375) days. Almost half of participants had a fall history before admission (45.5%) or lived with dementia (47.1%), while approximately one third had depression (36.7%) or cognitive impairment (32.9%). More than one fifth of the participants had anxiety (23.9%), cerebrovascular accident (23.7%) or diabetes mellitus (22.7%).

**Table 1:** Sample Characteristics for All Participants and by Place of Birth

	All sample (N = 5,628)	Australia-born (N = 4,086)	Overseas-born		
			All (N = 1,542)	Overseas English-speaking countries (N = 743)	Overseas non-English-speaking countries (N = 799)
**Male, n (%)**	1,896 (33.7)	1,304 (31.9)	592 (38.4)	287 (38.6)	305 (38.2)
**Age, median (IQR)**	86 (81–90)	86 (81–90)	85 (80–90)	85 (80–91)	85 (79–90)
**Age category, n (%)**					
65–79	1,186 (21.1)	808 (19.8)	378 (24.5)	174 (23.4)	204 (25.5)
80–84	1,139 (20.2)	798 (19.5)	341 (22.1)	157 (21.1)	184 (23.0)
85–89	1,636 (29.1)	1,243 (30.4)	393 (25.5)	184 (24.8)	209 (26.2)
≥ 90	1,667 (29.6)	1,237 (30.3)	430 (27.9)	228 (30.7)	202 (25.3)
**Place of birth, n (%)**					
Australia	4,086 (72.6)	4,086 (100.0)	–	–	–
Overseas	1,542 (27.4)	–	1,542 (100.0)	743 (100.0)	799 (100.0)
–Europe	984 (17.5)	–	984 (63.8)	437 (58.8)	547 (68.5)
–Non-Europe	558 (9.9)	–	558 (36.2)	306 (41.2)	252 (31.5)
**Length of stay (days), median (IQR)**	542 (69 − 1,375)	560 (78 − 1,418)	476 (55 − 1,259)	516 (70 − 1,217)	445 (45 − 1,288)
**Having fall history, n (%)**	2,562 (45.5)	1,884 (46.1)	678 (44.0)	337 (45.4)	341 (42.7)
**Health status, n (%)**					
Dementia	2,652 (47.1)	1,917 (46.9)	735 (47.7)	342 (46.0)	393 (49.2)
Depression	2,066 (36.7)	1,513 (37.0)	553 (35.9)	241 (32.4)	312 (39.1)
Cognitive impairment	1,853 (32.9)	1,368 (33.5)	485 (31.5)	236 (31.8)	249 (31.2)
Anxiety	1,344 (23.9)	1,007 (24.7)	337 (21.9)	150 (20.2)	187 (23.4)
Cerebrovascular accident	1,335 (23.7)	977 (23.9)	358 (23.2)	157 (21.3)	201 (25.2)
Diabetes mellitus	1,259 (22.4)	818 (20.0)	441 (28.6)	176 (23.7)	265 (33.2)
Visual impairment	913 (16.2)	685 (16.8)	228 (14.8)	116 (15.6)	112 (14.0)
Delirium	459 (8.2)	326 (8.0)	133 (8.6)	64 (8.6)	69 (8.6)
Parkinson’s disease	333 (5.9)	247 (6.1)	86 (5.6)	40 (5.4)	46 (5.8)

### Fall incidence rate by place of birth

**Fig. 2:**
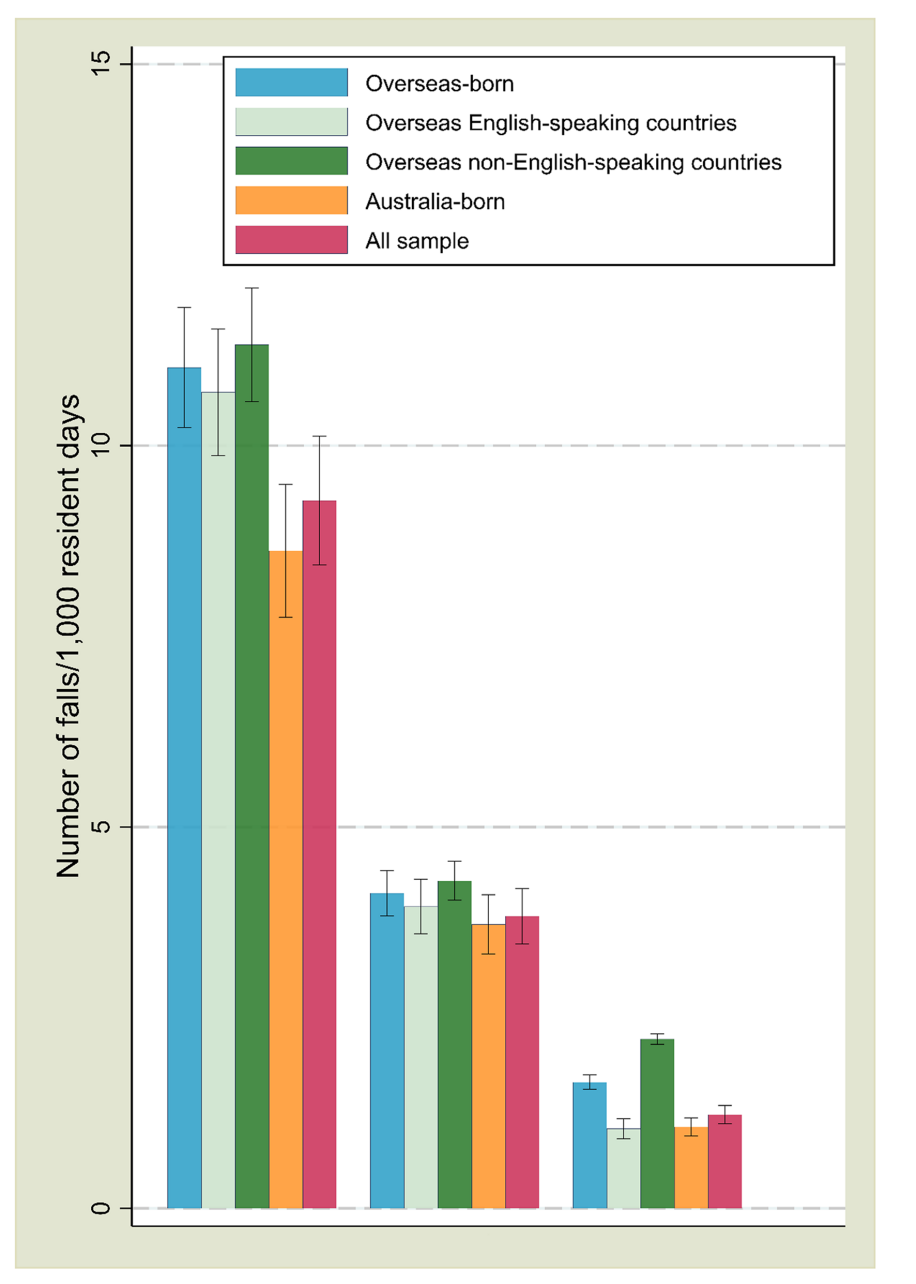
Incidence Rate of All Falls, Injurious Falls and Falls Requiring Hospitalisation by Place of Birth

Figure 2 presents the fall incidence rate for the whole sample and by place of birth. The falls incidence rates for all falls, injurious falls and falls requiring hospitalisation were 9.28 (95% CI = 8.43, 10.12), 3.83 (95% CI = 3.47, 4.20) and 1.23 (95% CI = 1.11, 1.35) incidents per 1,000 resident days, respectively. These figures were slightly higher among the overseas-born residents (11.02 [95% CI = 10.24, 11.81]; 4.13 [95% CI = 3.83, 4.43] and 1.65 [95% CI = 1.56, 1.75], respectively) than the Australia-born residents (8.62 [95% CI = 7.75, 9.49], 3.72 [95% CI = 3.33, 4.11] and 1.07 [95% CI = 0.95, 1.19], respectively). Within the overseas-born residents, these figures were relatively higher among those born in overseas non-English-speaking countries (11.32 [95% CI = 10.58, 12.07], 4.29 [95% CI = 4.04, 4.55] and 2.22 [95% CI = 2.15, 2.29], respectively) than those born in overseas English-speaking countries (10.70 [95% CI = 9.87, 11.53], 3.96 [95% CI = 3.60, 4.31] and 1.05 [95% CI = 0.93, 1.16], respectively).

### Association between fall incidents and place of birth

Table 2 presents the results of multilevel negative binomial regression to examine differences in fall risk between the Australia-born and the overseas-born residents. As shown, after controlling for the aforementioned covariates, the overseas-born residents as a whole had a significantly higher risk for all three falls groups (all falls: IRR = 1.278 [95% CI = 1.131, 1.443, p-value < 0.01]; injurious falls: IRR = 1.164 [95% CI = 1.013, 1.338, p-value = 0.03]; and falls requiring hospitalisation: IRR = 1.460 [95% CI = 1.199, 1.777, p-value < 0.01]) compared with the Australia-born residents.

**Table 2:** Fall Risk of the Overseas-born RACF Residents Compared with the Australia-born RACF Residents

	All falls	Injurious falls	Falls requiring hospitalisation
	IRR (95% CI)	IRR (95% CI)	IRR (95% CI)
**Place of birth (ref.=Australia)**			
–Overseas	1.278 (1.131,1.443)	1.164 (1.013,1.338)	1.460 (1.199,1.777)
**Age**	1.001 (0.993,1.008)	1.009 (1.000,1.017)	1.010 (0.998,1.022)
**Gender (ref.=female)**	1.726 (1.532,1.943)	1.898 (1.658,2.173)	1.602 (1.327,1.933)
**Fall history (ref.=no)**	1.249 (1.118,1.395)	1.231 (1.085,1.397)	1.107 (0.931,1.317)
**Entry type (ref.=respite)**			
–Permanent	0.563 (0.501,0.633)	0.557 (0.487,0.637)	0.546 (0.454,0.657)
**Dementia (ref.=no)**	1.630 (1.459,1.821)	1.422 (1.252,1.615)	1.416 (1.184,1.694)
**Depression (ref.=no)**	0.924 (0.819,1.044)	0.903 (0.787,1.037)	0.749 (0.616,0.910)
**Cognitive impairment (ref.=no)**	1.251 (1.114,1.404)	1.220 (1.067,1.394)	1.500 (1.242,1.811)
**Anxiety (ref.=no)**	0.913 (0.797,1.046)	0.963 (0.825,1.124)	1.005 (0.810,1.245)
**Cerebrovascular accident (ref.=no)**	1.203 (1.059,1.368)	1.384 (1.193,1.605)	1.296 (1.052,1.597)
**Diabetes mellitus (ref.=no)**	0.941 (0.826,1.072)	0.797 (0.686,0.926)	0.901 (0.731,1.111)
**Visual impairment (ref.=no)**	1.050 (0.907,1.215)	1.293 (1.090,1.533)	1.305 (1.025,1.662)
**Delirium (ref.=no)**	1.220 (1.000,1.488)	1.132 (0.904,1.417)	0.943 (0.693,1.282)
**Parkinson’s disease (ref.=no)**	1.844 (1.467,2.316)	1.675 (1.294,2.168)	1.339 (0.939,1.907)

Table 3 shows differences in fall risk between the Australia-born residents and the two subgroups of overseas-born residents. As shown, residents born in overseas English-speaking countries exhibited a significantly higher risk of all falls compared with their Australia-born counterparts (IRR = 1.259 [95% CI = 1.073, 1.478, p-value < 0.01]), though their risk of injurious falls (IRR = 1.192 [95% CI = 0.993, 1.432, p-value = 0.06]) and falls requiring hospitalisation (IRR = 1.126 [95% CI = 0.875, 1.448, p-value = 0.36]) did not significantly differ from those of Australia-born residents. Similarly, residents born in overseas non-English-speaking countries experienced a significantly higher risk of all falls than the Australia-born residents (IRR = 1.296 [95% CI = 1.106, 1.520, p-value < 0.01]) and had a higher risk of falls requiring hospitalisation compared with the Australia-born residents (IRR = 1.852 [95% CI = 1.431, 2.396, p-value < 0.01]). However, there were no differences in risk of injurious falls between the residents born in overseas non-English-speaking countries and those Australia-born (IRR = 1.136 [95% CI = 0.946, 1.364, p-value = 0.17]). Additionally, residents born in non-English-speaking countries experienced a significantly elevated risk of falls requiring hospitalisation (IRR = 1.645 [95% CI = 1.185, 2.282, p-value < 0.01]) compared to those born in English-speaking overseas countries, though there were no differences for all falls (IRR = 1.030 [95% CI = 0.837, 1.265, p-value = 0.78]) and injurious falls (IRR = 0.953 [95% CI = 0.751, 1.207, p-value = 0.69]).

**Table 3:** Fall Risk of Two Overseas-born Subgroups Compared with Australia-born RACF Residents

	All falls	Injurious falls	Falls requiring hospitalisation
	IRR (95% CI)	IRR (95% CI)	IRR (95% CI)
**Place of birth (ref.=Australia)**			
–Overseas English-speaking countries	1.259 (1.073,1.478)	1.192 (0.993,1.432)	1.126 (0.875,1.448)
–Overseas non-English-speaking countries	1.296 (1.106,1.520) (1.030 [0.837, 1.265]) ^**†**^	1.136 (0.946,1.364) (0.953 [0.751, 1.207]) ^**†**^	1.852 (1.431,2.396) (1.645 [1.185, 2.282]) ^**†**^
**Age**	1.001 (0.993,1.008)	1.009 (1.000,1.017)	1.009 (0.998,1.022)
**Gender (ref.=women)**	1.726 (1.533,1.944)	1.900 (1.659,2.175)	1.607 (1.333,1.938)
**Fall history (ref.=no)**	1.250 (1.119,1.396)	1.229 (1.083,1.395)	1.108 (0.932,1.317)
**Entry type (ref.=respite)**			
–Permanent	0.564 (0.502,0.633)	0.556 (0.486,0.636)	0.566 (0.470,0.681)
**Dementia (ref.=no)**	1.630 (1.459,1.821)	1.422 (1.252,1.616)	1.413 (1.182,1.690)
**Depression (ref.=no)**	0.924 (0.818,1.043)	0.904 (0.788,1.039)	0.751 (0.619,0.911)
**Cognitive impairment (ref.=no)**	1.251 (1.114,1.405)	1.219 (1.066,1.393)	1.505 (1.248,1.815)
**Anxiety (ref.=no)**	0.912 (0.796,1.045)	0.965 (0.826,1.126)	0.977 (0.788,1.210)
**Cerebrovascular accident (ref.=no)**	1.203 (1.059,1.368)	1.384 (1.193,1.605)	1.291 (1.049,1.588)
**Diabetes mellitus (ref.=no)**	0.939 (0.823,1.070)	0.800 (0.687,0.930)	0.860 (0.697,1.062)
**Visual impairment (ref.=no)**	1.050 (0.907,1.215)	1.294 (1.091,1.534)	1.318 (1.036,1.677)
**Delirium (ref.=no)**	1.220 (1.000,1.489)	1.130 (0.902,1.415)	0.949 (0.699,1.289)
**Parkinson’s disease (ref.=no)**	1.843 (1.467,2.316)	1.675 (1.294,2.168)	1.303 (0.916,1.854)

*Note: †, using the overseas-English-speaking-country-born residents as reference*

Examination of fall risks by country of birth, gender, age and admission type (Table 4) showed that males born overseas had a greater risk of falls for all categories compared to Australia-born male residents (all falls: IRR = 1.447 [95% CI = 1.174, 1.783, p-value < 0.01]; injurious falls: IRR = 1.366 [95% CI = 1.079, 1.729, p-value = 0.01]; and falls requiring hospitalisation: IRR = 2.382 [95% CI = 1.662, 3.413, p-value < 0.01]). There were no significant differences in falls risk for females by place of birth. Permanent residents born overseas had a higher risk of all falls compared to Australia-born permanent residents (IRR = 1.316 [95% CI = 1.146, 1.510, p-value < 0.01]). Overseas-born respite residents had a greater risk of all falls (IRR = 1.335 [95% CI = 1.069, 1.669, p-value = 0.01]) and falls requiring hospitalisation (IRR = 1.878 [95% CI = 1.261, 2.797, p-value < 0.01]) compared to Australia-born respite residents. No clear patterns were identified in relation to risk of falls and country of birth by age group. For full regression results presented in Table 4, please refer to Additional files 1.

**Table 4:** Fall Risk of Overseas-born RACF Residents vs. Australia-born, Stratified by Gender, Age and Admission Type

	Male	Female		Age ≤ 85	Age > 85		Permanent	Respite
	IRR (95% CI)	IRR (95% CI)		IRR (95% CI)	IRR (95% CI)		IRR (95% CI)	IRR (95% CI)
	All Falls							
**Place of birth (ref.=Australia)**								
–Overseas	1.447 (1.174,1.783)	1.144 (0.984,1.330)		1.321 (1.087,1.605)	1.235 (1.050,1.453)		1.316 (1.146,1.510)	1.335 (1.069,1.669)
	**Injurious Falls**							
**Place of birth (ref.=Australia)**								
–Overseas	1.366 (1.079,1.729)	1.027 (0.863,1.221)		1.391 (1.111,1.742)	0.952 (0.793,1.144)		1.130 (0.974,1.311)	1.292 (0.992,1.682)
	**Falls Requiring Hospitalisation**							
**Place of birth (ref.=Australia)**								
–Overseas	2.382 (1.662,3.413)	0.890 (0.708,1.118)		1.282 (0.955,1.720)	1.331 (1.019,1.738)		1.208 (0.992,1.471)	1.878 (1.261,2.797)

## Discussion

Populations are ageing and increasingly CALD in Australia and many other countries [23, 32]. In 2021, 17.6% of the Australian population was aged 65 and older and 27.6% of the Australian population was overseas-born [23]. Understanding how place of birth is associated with risk of falling potentially may assist in targeting prevention programs. Our study advances the knowledge of falls and place of birth by providing the first in-depth investigation into the patterns of fall risk by place of birth in the RACF setting. Our study shows that the overseas-born RACF residents were overall more susceptible to falls than their Australia-born counterparts, and that such differences in fall risk were more significant among men, respite residents and those born in overseas non-English-speaking countries. These findings provide a more granular understanding regarding the relationship between place of birth and falls. However, the study cannot shed light on the reasons for these differences.

This study demonstrates that immigrant status to Australia is associated with an increased fall risk among RACF residents. This finding is in contrast to previous Australian studies showing a lower fall risk for specific immigrant groups in the community-based setting [12, 15]. However, our results align with other studies indicating an adverse effect of immigration status and the post-migration environment on fall risk [16]. Previous studies have shown worse health outcomes (e.g., self-rated health, physical function and emotional health) [33–35] for older overseas-born populations compared with older native-born populations. Thus, the elevated risk of falls among the older overseas-born RACF residents might be explained by their overall frailer health outcomes than the native-born older adults. Specifically, during their acculturation process, immigrants might experience multiple negative life experience, such as limited access to health and medical resource and perceived discrimination, which are all hazardous to health [36–39]. For example, perceived discrimination might induce health problems (e.g., worse mental health status) among the immigrants [40]. The adverse effects of these negative life experiences might accumulate throughout the lifecycle and contribute to less favourable health outcomes in later life [18, 41]. Given that RACF residents are generally at very old age (e.g., median age at 86 in our study) and more fragile than community-dwelling older adults, the older immigrants living in RACFs might be vulnerable to the long-term adverse effect caused by acculturation-related stress and challenges, and hence, suffer less favourable health outcomes, such as falls, than the Australia-born RACF residents.

Results also show that the greater vulnerability to falls for the overseas-born RACF residents is more prominent among those born in overseas non-English-speaking countries compared with those born in overseas English-speaking countries. This finding highlights the role of language barrier in health outcomes and wellbeing of the overseas-born RACF residents. Previous studies had demonstrated that low language proficiency is significantly associated with immigrants’ lower income, reduced employability and limited access to medical and health resources [42], which might lead to less favourable health outcomes. Moreover, immigrants’ acquired ability of English might decrease during their ageing process. Previous evidence showed that reduced cognitive function or dementia might trigger a degeneration of the second language among bilingual individuals since maintaining multiple language abilities requires increased cognitive demands compared with a single language and is difficult to be sustained with impaired cognitive function and dementia [43, 44]. In our study, almost half (47.7%) of the overseas-born participants suffered dementia, which might cause a decrease in their English language capacity, and hence, expose them to extra fall risk. However, more explorations are needed to understand how acquired language ability changes during ageing process among the bilingual individuals and the corresponding consequences of such changes on health outcomes of older adults.

This study also demonstrated that the higher risk of falls of the overseas-born RACF residents was more significant among men. This is inconsistent with previous findings reporting a higher propensity of falls among women [1, 2, 5] but corresponds to some recent studies documenting a higher risk of falls among men [6, 45–48]. It is claimed that higher risk of falls among older women is compounded by female worse health conditions in old age, and if conditions of physical functionality and comorbidity status are controlled, men are actually more likely than women to fall [47, 48]. Therefore, given that RACF residents are generally in very old age and suffer deteriorated health conditions, male RACF residents might manifest a higher risk of falls. The greater probability of falls associated with male gender might be explained by male greater probability of engaging in physical activities and risky behaviour [49], which are associated with increased fall risk among the older adults. However, further investigations are needed to verify such assumption.

Another interesting finding is that the overseas-born RACF residents’ higher risk of falls was more prominent among respite residents compared with permanent residents. This intriguing finding corresponds to previous studies demonstrating a higher risk of adverse events, such as hospitalisation, for older adults recently admitted to RACFs compared with those having stayed for a long period [50, 51]. The heightened risk for respite residents might be explained by their maladjustment and unsettlement to the changes before and after RACF admission in relation to diets, routines, living environment and services, which require a certain amount of time to be adapt to. Such adaption might be relatively more difficult for the overseas-born RACF residents if there are accompanied with linguistical and cultural conflicts, which therefore elevate risk of falls.

### Policy implications

This study has important policy implications for improved fall prevention and management in the RACF setting for those residents born overseas. In particular, male and respite residents are at increased risk of fall. The diverse cultural and linguistical backgrounds of residents should be considered when developing fall prevention and intervention programs. Providing culturally and linguistically appropriate care service support, such as employing bilingual and bicultural staff and reflecting cultural diversity in fall intervention strategies (e.g., implementing some cultural practices such as Taichi), is suggested. In Australia, 35% of aged care workforce were from a CALD background in 2020 and this proportion is increasing [52]. However, the culturally appropriate service/care provision is generally only available for culturally dominant groups but tends to be lacking for those cultural minority groups. It would be critical to further increase the availability and accessibility of culturally and linguistically tailed care services in RACFs.

### Strengthens and limitations

We conducted a retrospective longitudinal cohort study on fall and place of birth in the RACF setting based on routinely collected data from 25 Australian RACFs. The strengthens of this study lie in its large sample size (i.e. 5,628 participants over more than five years) and its comprehensive measure of falls (risk of three types of falls gauged), which fortify the robustness of the results.

This study has several limitations. First, the data used were sourced from one aged care service provider in Sydney, New South Wales, Australia and therefore may not be reflective of other RACF populations. Second, while the analysis in this study has considered a broad range of covariates, some variables, such as level of general health and frailty that are useful to explain our results, have not been included in the analysis given data unavailability. Additionally, factors at organisational or environmental level, such as the location of facility, the number of staff and the availability of registered nurse, that might also importantly affect falls were also not controlled given the same reason. Future studies are suggested to include such variables if data are available. Third, even though we had categorised the overseas-born RACF residents into two subgroups, there are still considerable cultural and linguistic variations within each group. We were unable to further explore the variations of fall risk by place of birth within each group or by language preference of residents due to a lack of data. It would be of interest for future studies to examine more nuanced differentials of fall risk among different immigrant groups (e.g., by ethnicity or characteristics of country of origin [e.g., national income level]) if data are available. Fourth, migrants’ health outcomes might differ by duration of residence [18, 41], which is an important dimension of migrant health but has not been explored in this study. Specifically, migrants upon arrival might enjoy health advantage compared with the native-born population due to the selectivity of migration and the health requirement of the host society; however, such health advantage might be eroded during migrants acculturation process, resulting in converged health patterns between the native-born and migrants over time [53]. Therefore, the fall patterns of overseas-born RACF residents might vary according to their duration of residence. However, we are unable to examine this given lack of data. Further examination is needed to explore the fall pattern of overseas-born RACF residents by duration of residency.

## Conclusion

This study provides the first in-depth investigation into falls and place of birth in the RACF setting using a longitudinal cohort dataset in 25 RACFs Australia. Our analysis demonstrates that the overseas-born RACF residents experienced an overall higher fall risk than the Australia-born RACF residents, particularly among men, respite residents and those born in overseas non-English-speaking countries. The heightened fall risk associated with the overseas-born status highlights the importance of targeted fall prevention measures that are culturally and linguistically tailored for RACF residents.

## Electronic supplementary material

Below is the link to the electronic supplementary material.

Supplementary Material 1:
**Table S1.** Fall Risk of Overseas-born RACF Residents Compared with Australia-born RACF Residents, Male.
**Table S2.** Fall Risk of Overseas-born RACF Residents Compared with Australia-born RACF Residents, Female.
**Table S3.** Fall Risk of Overseas-born RACF Residents Compared with Australia-born RACF Residents, Age ≤ 85.
**Table S4.** Fall Risk of Overseas-born RACF Residents Compared with Australia-born RACF Residents, Age > 85.
**Table S5.** Fall Risk of Overseas-born RACF Residents Compared with Australia-born RACF Residents, Permanent Residents.
**Table S6.** Fall Risk of Overseas-born RACF Residents Compared with Australia-born RACF Residents, Respite Residents.

## Data Availability

The data that support the findings of this study are available from the aged care provider (Anglicare), but restrictions apply to the availability of these data, which were used under license for the current study, and so are not publicly available. Data are however available from the authors (Guogui Huang, guogui.huang@mq.edu.au) upon reasonable request and with permission of Anglicare.

## Abbreviations

RACF: residential aged care facility
CALD: culturally and linguistically diverse
CI: confidence interval
IRR: incidence rate ratios
IQR: interquartile range
OR: odd ratio.
