# Supplementary material for: The relationship between fall incidents and place of birth in residential aged care facilities: a retrospective longitudinal cohort study
Source: BMC Geriatr. 2023 Apr 28;23:257. doi: 10.1186/s12877-023-03954-7 (PMC10148446; doi:10.1186/s12877-023-03954-7)
Supplement: Supplementary file 1 — Supplementary Material 1: Table S1. Fall Risk of Overseas-born RACF Residents Compared with Australia-born RACF Residents, Male. Table S2. Fall Risk of Overseas-born RACF Residents Compared with Australia-born RACF Residents, Female. Table S3. Fall Risk of Overseas-born RACF Residents Compared with Australia-born RACF Residents, Age ≤ 85. Table S4. Fall Risk of Overseas-born RACF Residents Compared with Australia-born RACF Residents, Age > 85. Table S5. Fall Risk of Overseas-born RACF Residents Compared with Australia-born RACF Residents, Permanent Residents. Table S6. Fall Risk of Overseas-born RACF Residents Compared with Australia-born RACF Residents, Respite Residents. [file 12877_2023_3954_MOESM1_ESM.docx]

Supplementary Table S1 Fall Risk of Overseas-born RACF Residents Compared with Australia-born RACF Residents, Male

|  | **All Falls** | **Injurious Falls** | **Falls requiring Hospitalisation** |
| --- | --- | --- | --- |
|  | **IRR (95% CI)** | **IRR (95% CI)** | **IRR (95% CI)** |
| **Place of birth (ref.=Australia)** |  |  |  |
| –Overseas | 1.447(1.174,1.783) | 1.366(1.079,1.729) | 2.382(1.662,3.413) |
| **Age** | 1.012(0.998,1.026) | 1.021(1.006,1.037) | 1.055(1.031,1.080) |
| **Fall history (ref.=no)** | 1.518(1.247,1.848) | 1.460(1.168,1.824) | 1.194(0.849,1.678) |
| **Entry type (ref.=respite)** |  |  |  |
| –Permanent | 0.618(0.497,0.767) | 0.591(0.464,0.754) | 0.637(0.437,0.928) |
| **Dementia (ref.=no)** | 1.584(1.304,1.925) | 1.264(1.012,1.579) | 1.226(0.870,1.728) |
| **Depression (ref.=no)** | 0.693(0.552,0.869) | 0.712(0.552,0.918) | 0.704(0.476,1.041) |
| **Cognitive impairment (ref.=no)** | 1.319(1.069,1.627) | 0.990(0.780,1.257) | 1.669(1.171,2.380) |
| **Anxiety (ref.=no)** | 0.959(0.729,1.263) | 1.102(0.808,1.501) | 1.023(0.629,1.663) |
| **Cerebrovascular accident (ref.=no)** | 0.927(0.743,1.158) | 1.013(0.787,1.304) | 1.044(0.724,1.504) |
| **Diabetes mellitus (ref.=no)** | 0.830(0.670,1.028) | 0.850(0.665,1.085) | 1.112(0.758,1.629) |
| **Visual impairment (ref.=no)** | 0.920(0.698,1.212) | 0.962(0.703,1.316) | 0.659(0.410,1.059) |
| **Delirium (ref.=no)** | 0.964(0.680,1.368) | 1.057(0.705,1.585) | 0.751(0.398,1.419) |
| **Parkinson’s disease (ref.=no)** | 2.337(1.684,3.244) | 1.920(1.325,2.783) | 1.743(1.010,3.008) |

Supplementary Table S2 Fall Risk of Overseas-born RACF Residents Compared with Australia-born RACF Residents, Female

|  | **All Falls** | **Injurious Falls** | **Falls requiring Hospitalisation** |
| --- | --- | --- | --- |
|  | **IRR (95% CI)** | **IRR (95% CI)** | **IRR (95% CI)** |
| **Place of birth (ref.=Australia)** |  |  |  |
| –Overseas | 1.144(0.984,1.330) | 1.027(0.863,1.221) | 0.890(0.708,1.118) |
| **Age** | 1.000(0.991,1.009) | 1.007(0.997,1.018) | 0.991(0.978,1.005) |
| **Fall history (ref.=no)** | 1.209(1.055,1.385) | 1.227(1.050,1.433) | 1.188(0.974,1.449) |
| **Entry type (ref.=respite)** |  |  |  |
| –Permanent | 0.575(0.500,0.660) | 0.581(0.496,0.682) | 0.650(0.531,0.796) |
| **Dementia (ref.=no)** | 1.677(1.465,1.919) | 1.510(1.291,1.765) | 1.626(1.328,1.990) |
| **Depression (ref.=no)** | 1.053(0.914,1.214) | 1.025(0.872,1.206) | 0.864(0.698,1.070) |
| **Cognitive impairment (ref.=no)** | 1.215(1.056,1.398) | 1.357(1.153,1.595) | 1.514(1.223,1.875) |
| **Anxiety (ref.=no)** | 0.916(0.784,1.068) | 0.925(0.776,1.104) | 1.081(0.865,1.353) |
| **Cerebrovascular accident (ref.=no)** | 1.386(1.183,1.623) | 1.603(1.334,1.925) | 1.314(1.034,1.669) |
| **Diabetes mellitus (ref.=no)** | 1.042(0.882,1.230) | 0.797(0.658,0.966) | 0.941(0.729,1.214) |
| **Visual impairment (ref.=no)** | 1.125(0.945,1.340) | 1.294(1.053,1.590) | 1.365(1.043,1.786) |
| **Delirium (ref.=no)** | 1.347(1.059,1.713) | 1.170(0.892,1.535) | 1.084(0.768,1.529) |
| **Parkinson’s disease (ref.=no)** | 1.612(1.157,2.247) | 1.608(1.102,2.347) | 1.437(0.883,2.339) |

Supplementary Table S3 Fall Risk of Overseas-born RACF Residents Compared with Australia-born RACF Residents, Age ≤85

|  | **All Falls** | **Injurious Falls** | **Falls requiring Hospitalisation** |
| --- | --- | --- | --- |
|  | **IRR (95% CI)** | **IRR (95% CI)** | **IRR (95% CI)** |
| **Place of birth (ref.=Australia)** |  |  |  |
| –Overseas | 1.321(1.087,1.605) | 1.391(1.111,1.742) | 1.282(0.955,1.720) |
| **Age** | 0.995(0.978,1.012) | 1.006(0.987,1.025) | 1.006(0.981,1.032) |
| **Gender (ref.=women)** | 1.725(1.431,2.079) | 2.048(1.653,2.538) | 1.258(0.945,1.674) |
| **Fall history (ref.=no)** | 1.309(1.096,1.564) | 1.423(1.159,1.746) | 1.181(0.902,1.546) |
| **Entry type (ref.=respite)** |  |  |  |
| –Permanent | 0.512(0.424,0.618) | 0.455(0.366,0.565) | 0.481(0.362,0.638) |
| **Dementia (ref.=no)** | 1.876(1.577,2.231) | 1.582(1.289,1.940) | 2.170(1.643,2.866) |
| **Depression (ref.=no)** | 0.919(0.754,1.119) | 0.892(0.712,1.117) | 0.930(0.694,1.247) |
| **Cognitive impairment (ref.=no)** | 1.386(1.151,1.669) | 1.306(1.053,1.619) | 1.654(1.232,2.219) |
| **Anxiety (ref.=no)** | 0.988(0.795,1.228) | 1.103(0.864,1.408) | 0.974(0.695,1.364) |
| **Cerebrovascular accident (ref.=no)** | 1.099(0.893,1.352) | 1.313(1.027,1.678) | 1.185(0.853,1.647) |
| **Diabetes mellitus (ref.=no)** | 0.980(0.806,1.192) | 0.802(0.639,1.007) | 1.416(1.043,1.922) |
| **Visual impairment (ref.=no)** | 1.107(0.853,1.435) | 1.714(1.254,2.343) | 2.222(1.456,3.389) |
| **Delirium (ref.=no)** | 1.281(0.928,1.767) | 1.127(0.783,1.623) | 0.854(0.525,1.389) |
| **Parkinson’s disease (ref.=no)** | 1.973(1.454,2.679) | 1.812(1.274,2.577) | 1.879(1.187,2.975) |

Supplementary Table S4 Fall Risk of Overseas-born RACF Residents Compared with Australia-born RACF Residents, Age >85

|  | **All Falls** | **Injurious Falls** | **Falls requiring Hospitalisation** |
| --- | --- | --- | --- |
|  | **IRR (95% CI)** | **IRR (95% CI)** | **IRR (95% CI)** |
| **Place of birth (ref.=Australia)** |  |  |  |
| –Overseas | 1.235(1.050,1.453) | 0.952(0.793,1.144) | 1.331(1.019,1.738) |
| **Age** | 1.000(0.981,1.020) | 1.026(1.002,1.049) | 1.019(0.988,1.052) |
| **Gender (ref.=women)** | 1.721(1.473,2.012) | 1.888(1.588,2.245) | 2.029(1.585,2.598) |
| **Fall history (ref.=no)** | 1.278(1.107,1.474) | 1.264(1.077,1.484) | 1.243(0.991,1.560) |
| **Entry type (ref.=respite)** |  |  |  |
| –Permanent | 0.652(0.561,0.756) | 0.739(0.624,0.875) | 0.714(0.562,0.907) |
| **Dementia (ref.=no)** | 1.430(1.239,1.651) | 1.334(1.134,1.569) | 1.121(0.894,1.406) |
| **Depression (ref.=no)** | 0.982(0.840,1.148) | 1.016(0.853,1.210) | 0.760(0.590,0.980) |
| **Cognitive impairment (ref.=no)** | 1.135(0.977,1.319) | 1.077(0.910,1.275) | 1.351(1.068,1.708) |
| **Anxiety (ref.=no)** | 0.874(0.735,1.041) | 0.919(0.755,1.117) | 1.047(0.798,1.375) |
| **Cerebrovascular accident (ref.=no)** | 1.215(1.028,1.436) | 1.180(0.979,1.421) | 1.139(0.876,1.481) |
| **Diabetes mellitus (ref.=no)** | 0.959(0.802,1.147) | 0.885(0.722,1.084) | 0.709(0.535,0.939) |
| **Visual impairment (ref.=no)** | 0.971(0.812,1.161) | 0.880(0.719,1.077) | 0.659(0.491,0.884) |
| **Delirium (ref.=no)** | 1.273(0.984,1.646) | 1.205(0.907,1.602) | 1.084(0.728,1.614) |
| **Parkinson’s disease (ref.=no)** | 1.789(1.234,2.592) | 1.588(1.052,2.397) | 0.998(0.562,1.772) |

Supplementary Table S5 Fall Risk of Overseas-born RACF Residents Compared with Australia-born RACF Residents, Permanent Residents

|  | **All Falls** | **Injurious Falls** | **Falls requiring Hospitalisation** |
| --- | --- | --- | --- |
|  | **IRR (95% CI)** | **IRR (95% CI)** | **IRR (95% CI)** |
| **Place of birth (ref.=Australia)** |  |  |  |
| –Overseas | 1.316(1.146,1.510) | 1.130(0.974,1.311) | 1.208(0.992,1.471) |
| **Age** | 1.014(1.005,1.023) | 1.040(1.030,1.051) | 1.036(1.023,1.050) |
| **Gender (ref.=women)** | 1.790(1.567,2.046) | 1.828(1.586,2.105) | 1.360(1.128,1.640) |
| **Fall history (ref.=no)** | 1.322(1.168,1.497) | 1.404(1.228,1.605) | 1.238(1.037,1.479) |
| **Dementia (ref.=no)** | 1.337(1.184,1.510) | 1.340(1.174,1.529) | 1.206(1.012,1.437) |
| **Depression (ref.=no)** | 1.130(0.993,1.285) | 1.154(1.005,1.326) | 1.094(0.908,1.318) |
| **Cognitive impairment (ref.=no)** | 1.278(1.125,1.451) | 1.195(1.040,1.374) | 1.173(0.975,1.411) |
| **Anxiety (ref.=no)** | 0.929(0.805,1.070) | 0.980(0.841,1.143) | 0.993(0.811,1.216) |
| **Cerebrovascular accident (ref.=no)** | 1.107(0.965,1.269) | 1.192(1.029,1.381) | 0.903(0.741,1.101) |
| **Diabetes mellitus (ref.=no)** | 0.933(0.805,1.080) | 0.935(0.796,1.099) | 0.923(0.741,1.149) |
| **Visual impairment (ref.=no)** | 0.861(0.737,1.007) | 0.860(0.726,1.019) | 0.749(0.597,0.940) |
| **Delirium (ref.=no)** | 1.499(1.209,1.859) | 1.522(1.208,1.918) | 1.454(1.078,1.961) |
| **Parkinson’s disease (ref.=no)** | 1.625(1.258,2.101) | 2.042(1.551,2.690) | 1.536(1.066,2.212) |

Supplementary Table S6 Fall Risk of Overseas-born RACF Residents Compared with Australia-born RACF Residents, Respite Residents

|  | **All Falls** | **Injurious Falls** | **Falls requiring Hospitalisation** |
| --- | --- | --- | --- |
|  | **IRR (95% CI)** | **IRR (95% CI)** | **IRR (95% CI)** |
| **Place of birth (ref.=Australia)** |  |  |  |
| –Overseas | 1.335(1.069,1.669) | 1.292(0.992,1.682) | 1.878(1.261,2.797) |
| **Age** | 0.995(0.981,1.009) | 0.995(0.979,1.011) | 1.000(0.977,1.024) |
| **Gender (ref.=women)** | 1.681(1.351,2.090) | 2.034(1.566,2.641) | 1.562(1.064,2.293) |
| **Fall history (ref.=no)** | 1.199(0.975,1.473) | 1.169(0.918,1.489) | 1.055(0.750,1.483) |
| **Dementia (ref.=no)** | 1.976(1.601,2.439) | 1.489(1.156,1.917) | 1.712(1.174,2.497) |
| **Depression (ref.=no)** | 0.772(0.611,0.975) | 0.780(0.589,1.032) | 0.555(0.373,0.825) |
| **Cognitive impairment (ref.=no)** | 1.179(0.948,1.467) | 1.184(0.909,1.542) | 1.539(1.041,2.273) |
| **Anxiety (ref.=no)** | 0.883(0.678,1.150) | 0.933(0.681,1.276) | 0.906(0.573,1.432) |
| **Cerebrovascular accident (ref.=no)** | 1.239(0.966,1.590) | 1.335(0.980,1.818) | 1.411(0.908,2.193) |
| **Diabetes mellitus (ref.=no)** | 0.903(0.716,1.140) | 0.682(0.514,0.905) | 0.984(0.635,1.524) |
| **Visual impairment (ref.=no)** | 1.238(0.924,1.658) | 1.670(1.155,2.413) | 1.758(1.021,3.025) |
| **Delirium (ref.=no)** | 0.924(0.636,1.342) | 0.852(0.549,1.321) | 0.616(0.330,1.152) |
| **Parkinson’s disease (ref.=no)** | 2.071(1.367,3.138) | 1.339(0.825,2.172) | 1.148(0.585,2.253) |
